# *trans*-Cinnamaldehyde Reverses Depressive-Like Behaviors in Chronic Unpredictable Mild Stress Rats by Inhibiting NF-*κ*B/NLRP3 Inflammasome Pathway

**DOI:** 10.1155/2020/4572185

**Published:** 2020-02-28

**Authors:** Meng Wang, Shuguang Yan, Yongxue Zhou, Pei Xie

**Affiliations:** ^1^Chengdu University of TCM, Chengdu 610075, China; ^2^Shanxi University of Chinese Medicines, Xianyang 712046, China

## Abstract

*trans*-Cinnamaldehyde (TCA) is the main active component extracted from *Cinnamomum cassia* (*C. cassia*), which has many pharmacological effects, such as anti-inflammation, lowering blood glucose, and improving nerve function. However, there is no report of TCA in the treatment of depression. The purpose of this study was to investigate the antidepressant-like effect of TCA and the mechanism of NF kappa B (NF-*κ*B) pathway and NLRP3 inflammasome inhibition by TCA. We divided 40 rats into the control group, CUMS group, FLU group, and the TCA group. The activation of the NF-*κ*B pathway and NLRP3 inflammasome in prefrontal cortex and hippocampus of rats in each group was observed. After the treatments with FLU and TCA, the sucrose consumptions in rats increased significantly and the immobility time in forced swimming was decreased significantly compared to the CUMS group. The expression of TLR4, NF-*κ*B-1, p-p65, TNF-*α*, NLRP3, ASC, caspase-1, IL-1*β*, and IL-18 proteins in prefrontal cortex and hippocampus was decreased, and the expression of IL-1*β*, IL-18, and TNF-*α* in serum was downregulated compared to the CUMS group. Similar to FLU, TCA reverses the depression-like behaviors in rats, which indicates that TCA has a significant antidepressant-like effect. The mechanism of the antidepressant property of TCA might be that it inhibits the activation of the NF-*κ*B pathway and NLRP3 inflammasome in the prefrontal cortex and hippocampus of CUMS rats.

## 1. Introduction

Depression, a major problem for public health, is a common mental disease that mainly manifests as lowered mood, slow thought process, decreased will activity, etc. Over 300 million people worldwide suffer from depression, which is predicted to be the largest disease in the world by 2030 [1, 2]. Depression is often associated with other chronic diseases due to its complex pathogenesis [3]. Depression poses great challenges to clinical practitioners, and more than 30% of patients do not receive effective treatments [4]. Thus, safe and effective therapies of depression are urgently needed.

In recent years, cytokines have been focused on explaining the pathophysiology of depression. The complex interactions between depression and release of proinflammatory factors caused by immune activation have been described [5, 6]. Cytokines directly act on neurons and supporting cells through the blood-brain barrier and then affect the brain's function and cognition [7]. The NLR family, pyrin domain-containing 3 (NLRP3) inflammasome is an intracellular polyprotein that is composed of NLRP3, apoptosis-associated speck-like protein (ASC), and procaspase-1. NLRP3 inflammasome is involved in many innate immune processes related to infection, inflammation, and autoimmunity [8]. NLRP3 inflammasome is a bridge between immune activation and stress exposure; the latter promotes the development of depression through the activation of NLRP3 inflammasome [9, 10]. The interleukin-1 beta (IL-1*β*) and IL-18 levels are increased in the peripheral blood monocytes of patients with major depression due to the activation of NLRP3 inflammasome [11]. The activation of NLRP3 inflammasome in microglia mediates IL-1*β*-related inflammatory response in the prefrontal cortex of depressed rats [12]. The protein of nuclear factor kappa-light-chain enhancer (NF-*κ*B) is activated in the hippocampus of depressed mice; however, the activation of NF-*κ*B is hindered when the NLRP3 gene is knocked out [13]. Thus, NLRP3 inflammasome is an inflammatory target of depression [14]. Inhibiting the activation of NLRP3 inflammasome plays an important role in the prevention and treatment of depression.

TCA is a main active component in *Cinnamomum cassia* (*C. cassia*), which exhibits a variety of pharmacological effects, such as anti-inflammatory, antitumor, hypoglycemia, and enhanced obesity [15–18]. TCA has a certain anti-neuroinflammatory effect, and its protective effect on dopaminergic neurons may be related to the inhibition of inflammation [19]. TCA inhibits microglia activation and inflammatory response induced by lipopolysaccharide (LPS) [20]. TCA reduces neuroinflammation via inhibiting NF-*κ*B pathway [21]. TCA should be considered as a potential drug for the treatment of neuroinflammation and neurodegenerative diseases [21].

The pathogenesis of depression is closely related to neuroinflammation, and the anti-inflammatory activity of TCA is related to its inhibition of the activation of NLRP3 inflammasome [22]. Importantly, the antidepressant-like effect of TCA has not been reported. Our previous study found that TCA reversed the depressive-like behaviors on the chronic unpredictable mild stress (CUMS) rats. However, it is not clear how TCA influences the activation of NLRP3 inflammasome and NF-*κ*B pathway to contribute to the neuroinflammation in CUMS rats. We hypothesize that TCA may treat depression via inhibiting the activation of NLRP3 inflammasome and NF-*κ*B pathway. In this study, we evaluated the neuroprotective effect and the anti-neuroinflammatory mechanism of TCA on CUMS rats.

## 2. Materials and Methods

### 2.1. Reagents

TCA (Cas No. 230835198, purity ≥98%) was purchased from Baoji Chenguang Biotechnology Co., Ltd. (Baoji China). Fluoxetine Hydrochloride (FLU, Cas No. 56296-78-7) was provided by Sigma-Aldrich Co., Ltd. (USA). Membrane/cytoplasm/nucleus-protein extraction kit (BB-3104) was purchased from Shanghai Bestbio Biotechnology Co., Ltd. (Shanghai China). Trizol Invitrogen (15596026), DNase/RNase-Free Water (R1600), BCA Protein Assay Kit (Cat # PC 0020), and toluidine blue dye solution (G3668) were supplied by Solarbio Science &Technology Co., Ltd. (Beijing China). Anti-TMS1 (ASC, ab175449), anti-caspase-1 (ab1872), anti-NLRP3 (ab214185), anti-IL-18 (ab191860), and anti-IL-1*β* (ab9722) antibodies were obtained from Abcam Biotechnology Co., Ltd. (Cambridge UK). Anti-GAPDH (bs-2188R), Anti-Lamin B (bs-1840R), anti-NF-*κ*B-1 (bs-1194R), anti-p-NF-*κ*B-1 (bs-3544R), anti-NF-*κ*B p65 (p65 bs-0465R), anti-p-NF-*κ*B p65 (p-p65 bs-0982R), anti-I*κ*B*α* (bs-1287R), anti-p-I*κ*B*α* (bs-5515R), anti-TLR4 (bs-20594R), and anti-tumor necrosis factor-alpha (TNF-*α*, bs-2081R) antibodies were provided by Bioss Biotechnology Co., Ltd. (Beijing China). Gene primers were provided by Sangon biological engineering co., Ltd. (Shanghai China); the primer sequences were as follows: *β*-actin: forward: 5′ cta agg cca acc gtg aaa ag3′, reverse: 5′tac atg gct ggg gtg ttg a3′; TLR-4: forward: 5′gct gcc aac atc atc cag gaa gg3′, reverse: 5′tga tgc cag agc ggc tac tca g3′; NF-*κ*B p65 (p65): forward: 5′ggc ttc tat gag gct gaa ctc tgc3′, reverse: 5′ctt gct cca ggt ctc gct tct tc3′; NF-*κ*B-1: forward: 5′tgt ggt gga gga ctt gct gag3′, reverse: 5′agt gct gcc ttg ctg ttc ttg ag3′; I*κ*B*α*: forward: 5′ggt ctc gct cct gtt gaa gtg tg3′, reverse: 5′tcc gtg tca tag ctc tcc tca tcc3′; TNF-*α*: forward: 5′atg tct cag cct ctt ctc att c3′, reverse: 5′gct tgt cac tcg aat ttt gag a3′. RT Master Mix (RR036A) and TB Green Premix Ex Taq II (RR820A) were purchased from TaKaRa Bio Group (Dalian China). IL-18 enzyme-linked immunosorbent assay (ELISA) kit (EK0592) was purchased from BOSTER Biological Engineering Co., Ltd. (Wuhan China). The IL-1*β* ELISA kit (70-EK301B) and TNF-*α* ELISA kit (70-EK382) were obtained from MULTISCIENCES Biotechnology Co., Ltd. (Hangzhou China).

### 2.2. Animals

**Figure 1:**
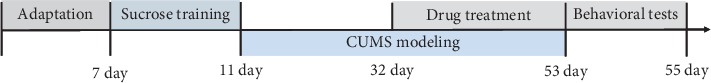
Experimental design. Timeline of animal experiments, chronic unpredictable mild stress (CUMS) procedure, drugs administering, and behavioral tests including sucrose preference test (SPT) and forced swim test (FST).

Forty male Sprague-Dawley rats (6-7 weeks old) weighing 180 ± 20 g were provided by the Animal Experimental Center of Xi'an Jiaotong University (P. R. China, certificate no. SCXK (Shan) 2017–003). The rats were housed in a controlled environment (temperature: 23 ± 2°C, lights: 8:00 a.m. to 8:00 p.m., humidity: 55 ± 5%). During 7-day adaptation period, rats were provided a standard diet and water free. All animal experimental protocols were approved by the guidelines of Shaanxi University of traditional Chinese Medicine Animal Ethics Committee.  Figure 1 shows the process of animal experiments.

### 2.3. Sucrose Preference Test (SPT)

After 7 days of adaptation, the rats were trained in sugar water intake and the baseline sucrose preference was determined. Rats in each cage were given 2 bottles of 1% sucrose solution for 24 h. The rats were given both 1% sucrose solution and water for another 24 h. After 24 h of water deprivation, the rats were given both sucrose solution and water for 1 h. Sucrose preference = weight of sucrose solution intake/(weight of sucrose solution intake + weight of water intake). The position of the bottle was changed in the middle of SPT.

### 2.4. CUMS Protocol and Drug Administration

After the determination of baseline sucrose preference, 10 rats were randomly assigned to the control group. Other rats were exposed to the CUMS procedure for 3 weeks. In brief, it was a randomized schedule that contained various stressors. The stressors are shown in Table 1. After CUMS procedure, the rats were randomly divided into 3 groups according to the body weight and sucrose preference: model group (*n* = 10), FLU group (*n* = 10), and TCA group (*n* = 10). FLU (10 mg/kg) and TCA (10 mg/kg) were diluted in sterile water, respectively. All drugs were orally administrated every day for 3 weeks. During treatment, the rats continued to receive stressors. The SPT was conducted once a week during the CUMS protocol and drug administration at a fixed time. Throughout the procedure, rats in the control group did not receive any stressor.

### 2.5. Forced Swimming Test (FST)

The FST was carried out on the rats at day 55. Each rat was individually put into a glass bucket (20 cm in diameter ∗ 40 cm high) containing a 25 cm depth of water (25 ± 1°C) for 6 min. During the test, the rats were allowed 1 min to adapt, and the immobility time of the rats was recorded during the final 5 min. To avoid olfactory cues, we changed the water after each swimming cycle.

### 2.6. Preparation of Tissue Specimen

After the final day of FST, rats were sacrificed under anesthesia with pentobarbital. The blood was collected from the abdominal aorta and coagulated for 30 min at 25°C. The supernatant serum (3500 rpm, 15 min) was separated and stored at −80°C. Rats were rapidly perfused with physiologic saline through the left cardiac ventricle. The brains of 7 rats in each group were taken out and placed in ice immediately. Five hippocampus and prefrontal cortex samples were carefully stripped off and stored at −80°C for further real-time quantitative polymerase chain reaction (qPCR) and Western Blot procedures. Two hippocampus samples were applied to observe the cell ultrastructure.

### 2.7. Nissl Staining

After anesthetizing, 3 rats from each group were rapidly perfused with physiologic saline followed by 4% paraformaldehyde. The brains were removed from the ice, fixed in 4% paraformaldehyde for 24 h, and embedded in paraffin for further staining. The fixed brains were cut into 5 *μ*m thick sections from the hippocampal area. Paraffin sections were dewaxed with xylene (10 min ∗ 2), hydrated by gradient ethanol (100%, 95%, 70%; 5 min), and washed with distilled water (5 min ∗ 3). After Nissl staining solution was preheated to 37°C, the treated tissues were stained with it for 5 min. After washing with distilled water, the tissue sections were dehydrated with 100% ethanol (5 min ∗ 2), made transparent with xylene (5 min ∗ 2), and sealed with neutral gum. The slides were observed under a light microscope.

### 2.8. Cell Ultrastructure

Ultrastructure and mitochondrial morphology of pyramidal cells in the hippocampal CA3 region was observed by transmission electron microscope (TEM). The tissues of the hippocampal CA3 region were cut into small pieces (1 ∗ 1∗ 1 mm), fixed in 2.5% glutaraldehyde (4°C, 3 h), soaked in 0.1 m phosphate buffer (30 min), fixed in 1% osmium tetroxide fixation solution (4°C, 3 h), and washed with 0.1 m phosphate buffer (10 min) and dehydrated with ethanol gradient dehydration: 30% ethanol (10 min), 50% ethanol (10 min), 70% ethanol (10 min), 70% ethanol acetic acid uranium peroxide block (2 h), 90% ethanol (10 min ∗ 2), and 100% ethanol (10 min ∗ 3). Tissues were replaced by oxy-propane (10 min) and soaked and embedded by epoxy resin Epon812. After polymerization, the semi-ultra-thin slices were made into 1-2 *μ*m. The slices were stained by methylene blue, located under optical microscope, sliced (50-70 nm) with UC7 ultra-thin slicer (German Leica Co., Ltd.), and stained with uranium acetate and lead citrate. The ultrastructure was observed under H-7650 transmission electron microscope (HITACHI Co., Ltd.).

### 2.9. ELISA Detection

The levels of IL-1*β*, IL-18, and TNF-*α* proteins were determined by ELISA kit. The ELISA tests were carried out according to the manufacturer's instructions. The levels of IL-1*β*, IL-18, and TNF-*α* proteins in serum were calculated according to the standard curve and dilution multiple.

### 2.10. q-PCR

The 100 mg tissue samples with 1 ml Trizol were fully homogenized, and the supernatant was collected into another EP tube after centrifuging (12000 rpm, 5 min, 4°C). 200 *μ*l chloroform was added in EP tube and mixed for 15 min at room temperature. After centrifuging (12000 rpm, 5 min, 4°C), the supernatant was collected into another EP tube. 0.5 ml isopropanol was added and placed for 10 min at room temperature. After centrifuging (12000 rpm, 10 min, 4°C), the RNA was submerged at the bottom of the tube. After washing by ethanol for 2 times, RNA was drying at room temperature and dissolving with 50 *μ*l DNase/RNase-free water. Reverse transcription and qPCR processes followed the instructions. In our study, the genes were as follows: TLR-4, NF-*κ*B p65 (p65), NF-*κ*B-1, I*κ*B*α*, and TNF-*α* were detected.

### 2.11. Western Blot

The 100 mg tissue samples were placed in the EP tube. Tissue samples were homogenized in RIPA buffer (containing protease inhibitor and phosphatase inhibitor) for 30 min. The supernatant was collected after centrifuging at 12000 rpm for 15 min at 4°C, and the concentrations of protein were determined with BCA kits. The protein was stored at −80°C after denaturation. Equal protein samples from hippocampus or prefrontal cortex were separated through 12% SDS-PAGE gel (concentrated gel 80v 30 min, separation gel 100v 90 min) and transferred to a polyvinylidene difluoride (PVDF) membrane (200 mA, 1-2 h). After blocking with Tris-buffered saline containing 0.1% Tween 20 (TBST), 5% fat-free milk (room temperature for 2 h or 4°C overnight), the membranes were incubated with specific primary antibodies (4°C overnight). After washing with TBST (4 times, per 5 min), the membranes were incubated with a second antibody (room temperature for 1 h) and washed with TBST (4 times, per 5 min). The bands were detected with an enhanced chemiluminescence (ECL) detection system and the values of the bands were analyzed by ImageJ software.

### 2.12. Statistical Analysis

Data were expressed as mean ± S.E.M. and analyzed using analysis of variance (one-way ANOVA). Significance was determined based on the Bonferroni test. The difference was considered to be significant when *p* < 0.05. All data were analyzed by SPSS 23.0 software.

## 3. Results

### 3.1. TCA Increases Body Weight of CUMS Rats

**Figure 2:**
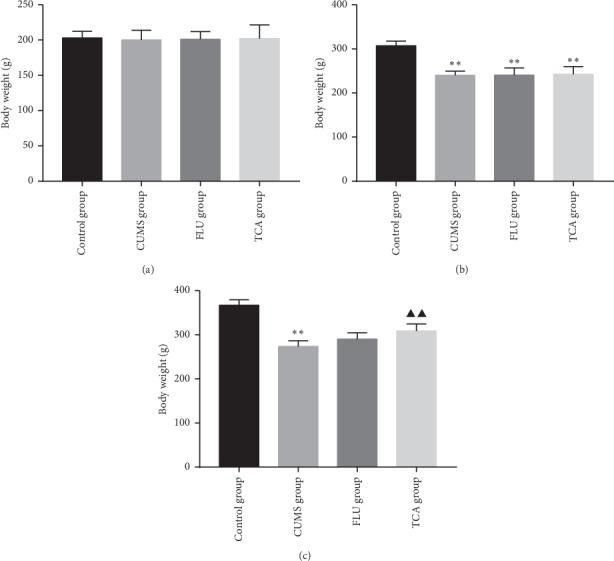
Body weight: (a) baseline body weight before CUMS procedure; (b) body weight of rats in each group after CUMS procedure; (c) body weight of rats after treatment. ^*∗∗*^*p* < 0.01 comparison with the control group, ^▲▲^*p* < 0.01 comparison with the CUMS group.

Before establishing CUMS, the body weight of rats in each group was approximately the same (Figure 2(a)). After 3 weeks of establishing the model, rats in each group exhibited a significant decline in body weight increase as compared to the control group (*p* < 0.01, *p* < 0.01, *p* < 0.01Figure 2(b)). After 3 weeks of TCA (10 mg/kg) treatment, the body weight increase was significantly higher (*p* < 0.01Figure 2(c)).

### 3.2. TCA Increases Sucrose Consumption in CUMS Rats

**Figure 3:**
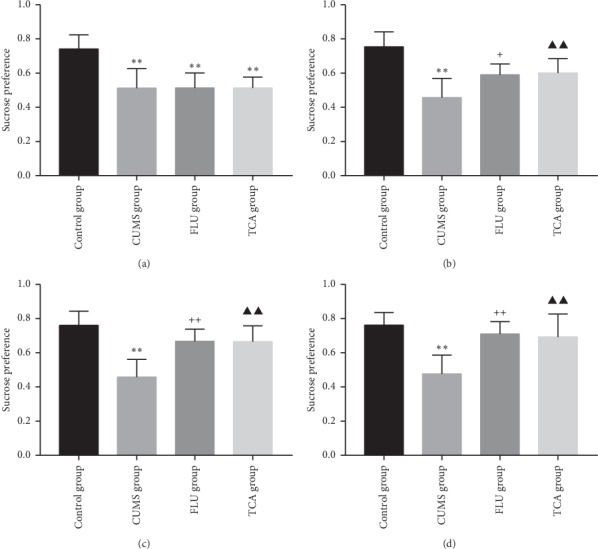
Sucrose preference of rats in each group: (a) after CUMS procedure; (b) after 1-week treatment; (c) after 2-week treatment; (d): sucrose preference of rats after 3-week treatment. ^*∗∗*^*p* < 0.01 comparison with the control group, ^+^*p* < 0.05 indicates comparison with the CUMS group, ^++^*p* < 0.01 comparison with the CUMS group, ^▲▲^*p* < 0.01 comparison with the CUMS group.

Figure 3 shows the effect of TCA on sucrose preference of CUMS rats. After establishing CUMS, the sucrose consumption of rats in each group decreased significantly compared to the control group (*p* < 0.01, *p* < 0.01, *p* < 0.01Figure 3(a)). Except for the control group, there was no significant difference in sucrose preference among the other groups. After 1-week treatment of FLU (10 mg/kg) and TCA (10 mg/kg) separately, the sucrose preference increased significantly (*p* < 0.05, *p* < 0.01Figure 3(b)). After 2- and 3-week treatment of FLU and TCA separately, the sucrose preference of rats increased (*p* < 0.01, *p* < 0.01 Figures 3(c) and 3(d)).

### 3.3. TCA Attenuates Depressive-Like Behaviors in CUMS Rats

**Figure 4:**
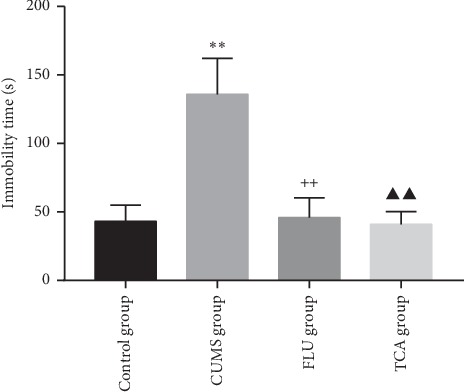
Immobility time of forced swimming of rats after 3-week treatment. ^*∗∗*^*p* < 0.01 indicates comparison with the control group, ^++^*p* < 0.01 comparison with the CUMS group, ^▲▲^*p* < 0.01 comparison with the CUMS group.

As shown in Figure 4, the immobility time of rats in the CUMS group was significantly increased (*p* < 0.01). After treatment with FLU and TCA for 3 weeks separately, the immobility time of rats was reduced (*p* < 0.01, *p* < 0.01) as compared to the CUMS group.

### 3.4. Results of Nissl Staining

**Figure 5:**
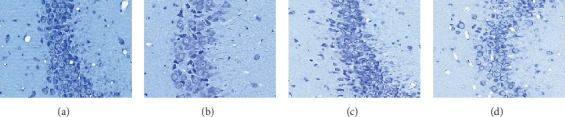
Nissl staining of hippocampal pyramidal cells in CA3. In the CUMS group, the number of Nissl corpuscles in neuron cells of hippocampus in CA3 was obviously less than that in the control group; however, FLU and TCA markedly increased the number of Nissl corpuscles. (a) Control group (200x), (b) CUMS group (200x), (c) FLU group (200x), (d) TCA group (200x).

Figure 5 shows the results of Nissl staining of hippocampal neurons in CA3. In the control group, the neurons in the hippocampal CA3 were neatly arranged and dense, the cell bodies were full, the staining was deep, the nucleus was large and round, and the Nissl corpuscles were rich. The number of neurons in the hippocampal CA3 region of the model rats was rare, the morphology was irregular, the staining was shallow, the cell body atrophied, and the Nissl corpuscles were rare. In FLU group, the number of neurons in hippocampal CA3 recovered well, the number of neurons was significantly increased, the morphology was regular, the cell body was fuller, the staining was deeper, the nucleus was larger and round, and the number of Nissl corpuscles was significantly increased. The number of neurons in CA3 of hippocampus in TCA group was significantly more than that in CUMS group, the shape was more regular, the arrangement was neater, and the number of Nissl corpuscles increased.

### 3.5. TCA Affects Ultrastructure of Pyramidal Cells in CA3 of CUMS Rats

**Figure 6:**
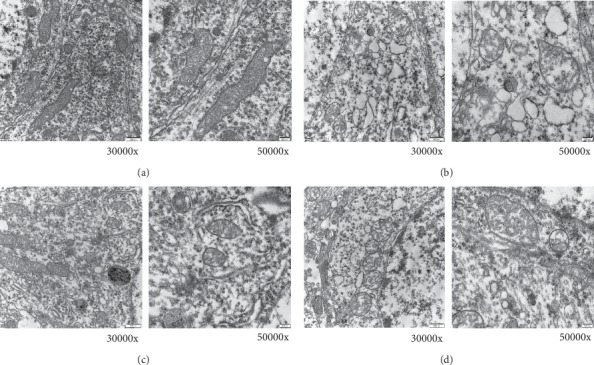
The ultrastructure of pyramidal cells in CA3. In the control group, the cell structure was clear and the mitochondria were round or rod-shaped. In CUMS group, the cells and the mitochondria were swollen; the crest decreased. In FLU group, the cell structure was clear, the mitochondria were slightly swollen, and the crest was clear. In the TCA group, the cell structure was better, the swelling degree was lighter, and the mitochondrial crest was clear. (a) Control group, (b) CUMS group, (c) FLU group, (d) TCA group.

Figure 6 shows the ultrastructure of pyramidal cells in CA3. In the control group, there were a large number of cells with clear cell structure, round nucleus, abundant organelle, and round or rod-shaped mitochondria in the cells, mitochondrial crest was found to be clear and developed rough endoplasmic, and ribosome and Golgi complex were also common. In the CUMS group, the cells were swollen, with basic clear cell structure. The chromatin in the nucleus was loose, the mitochondria swelled, the crest decreased or even disappeared, and some of the mitochondria showed vacuole-like changes. In the FLU group, the cells were dense, the cell structure was clearer, and cell swelling was lighter than that in the CUMS group. The mitochondria in the cytoplasm were slightly swollen and the mitochondrial crest was clear. Similar to FLU group, the TCA group had dense cells, better cell structure, and lighter cell swelling than that in the CUMS group. Slight swelling of the mitochondria and the cell swelling in the TCA group were less than those in CUMS group.

### 3.6. TCA Blocks NF-*κ*B Pathway of Prefrontal Cortex at mRNA Level in CUMS Rats

**Figure 7:**
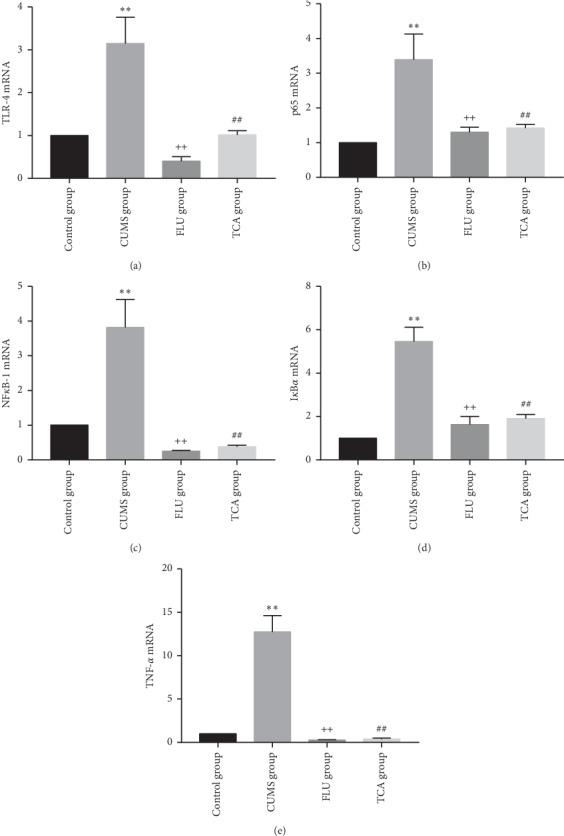
The effect of TCA on NF-*κ*B pathway of prefrontal cortex at mRNA level. After 3 weeks of treatments, the relative expressions of mRNA of TLR-4 (a), p65 (b), NF-*κ*B-1 (c), I*κ*B*α* (d), and TNF-*α* (e) in the prefrontal cortex of rats were tested. ^*∗∗*^*p* < 0.01 comparison with the control group, ^++^*p* < 0.01 comparison with the CUMS group, ^##^*p* < 0.01 comparison with the CUMS group.

Figure 7 shows the effect of TCA on NF-*κ*B pathway at mRNA level of prefrontal cortex in CUMS rats. Relative expression of mRNAs of TLR-4, p65, NF-*κ*B-1, I*κ*B*α*, and TNF-*α* in prefrontal cortex was significantly higher in the CUMS group than that in the control group (*p* < 0.01, *p* < 0.01, *p* < 0.01, *p* < 0.01, *p* < 0.01Figure 7(a) through Figure 7(e)). NF-*κ*B pathway mRNAs were downregulated by TCA and FLU after 3 weeks of treatment (*p* < 0.01, *p* < 0.01, *p* < 0.01, *p* < 0.01, *p* < 0.01Figure 7(a) through Figure 7(e)).

### 3.7. TCA Blocks NF-*κ*B Pathway of Prefrontal Cortex in CUMS Rats at Protein Level

**Figure 8:**
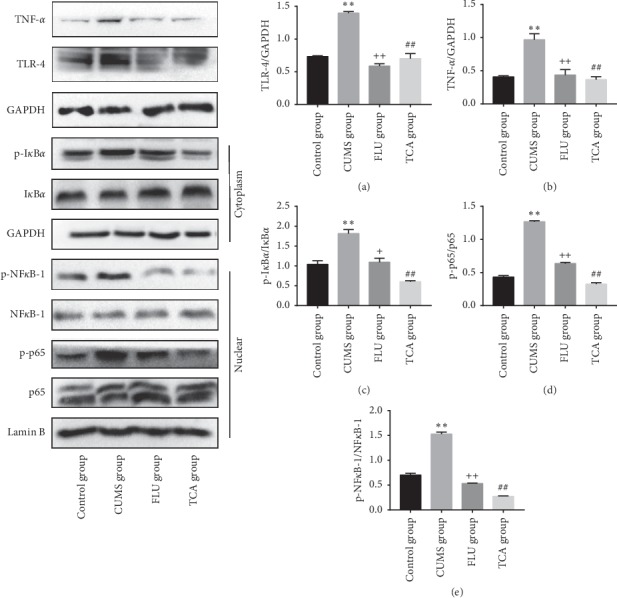
The effect of TCA on NF-*κ*B pathway of prefrontal cortex at protein level. The protein expressions of TLR-4 (a), TNF-*α* (b), p-I*κ*B*α* (c), p-p65 (d), and p-NF-*κ*B-1 (e) in the prefrontal cortex of rats were tested. ^*∗∗*^*p* < 0.01 comparison with the control group, ^+^ *p* < 0.05 comparison with the CUMS group, ^++^*p* < 0.01 comparison with the CUMS group, ^##^*p* < 0.01 comparison with the CUMS group.

The changes of NF-*κ*B pathway in prefrontal cortex are shown in Figure 8. CUMS significantly upregulated the levels of TLR-4 (*p* < 0.01Figure 8(a)) and TNF-*α* (*p* < 0.01Figure 8(b)). The protein level of p-IKB*α* (*p* < 0.01Figure 8(c)) in cytoplasm and the protein levels of p-p65 (*p* < 0.01Figure 8(d)) and p-NF-*κ*B-1 (*p* < 0.01Figure 8(e)) in nucleus were also upregulated. After 3-week treatment of TCA, the levels of TLR-4 (*p* < 0.01, *p* < 0.01Figure 8(a)) and TNF-*α* (*p* < 0.01, *p* < 0.01Figure 8(b)) were decreased. At the same time, the expression of p-IKB*α* (*p* < 0.01, *p* < 0.01Figure 8(c)) in cytoplasm and p-p65 (*p* < 0.01, *p* < 0.01Figure 8(d)) and p-NF-*κ*B-1 (*p* < 0.01, *p* < 0.01Figure 8(e)) in nucleus was downregulated.

### 3.8. TCA Reduces the NLRP3 Inflammasome Activation of the Prefrontal Cortex

**Figure 9:**
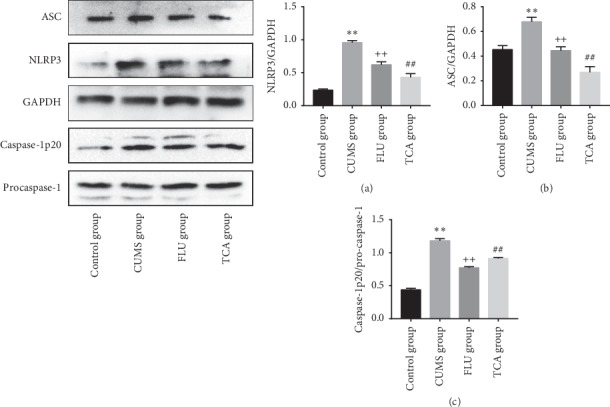
The effect of TCA on NLRP3 inflammasome of prefrontal cortex. The expressions of NLRP3 (a), ASC (b), and caspase-1 p20 (c) in the prefrontal cortex of rats were tested after treatments. ^*∗∗*^*p* < 0.01 comparison with the control group, ^++^*p* < 0.01 comparison with the CUMS group, ^##^*p* < 0.01 comparison with the CUMS group.

As shown in Figure 9, the NLRP3 inflammasome related proteins NLRP3 (*p* < 0.01Figure 9(a)), ASC (*p* < 0.01Figure 9(b)), and caspase-1 p20 (*p* < 0.01Figure 9(c)) were elevated in the prefrontal cortex of rats after establishing CUMS. Compared to the CUMS group, the activation of NLRP3 inflammasome (*p* < 0.01, *p* < 0.01Figure 9(a) through Figure 9(c)) was suppressed after 3 weeks of FLU and TCA separate treatment.

### 3.9. TCA Blocks the Expression of Inflammatory Factors in the Prefrontal Cortex

**Figure 10:**
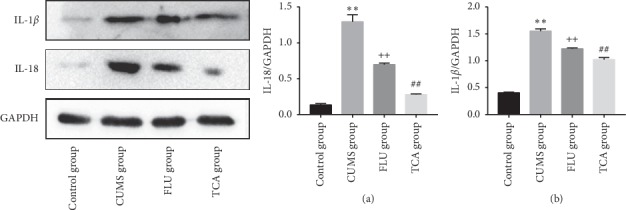
The effect of TCA on inflammatory factors of prefrontal cortex. The expressions of IL-18 (a) and IL-1*β* (b) in prefrontal cortex were tested. ^*∗∗*^*p* < 0.01 comparison with the control group, ^++^*p* < 0.01 comparison with the CUMS group, ^##^*p* < 0.01 comparison with the CUMS group.

To further investigate the effect of TCA on the activation of the NLRP3 inflammasome, we detected the downstream inflammatory factors IL-18 and IL-1*β*. The expression of IL-18 (*p* < 0.01Figure 10(a)) and IL-1*β* (*p* < 0.01Figure 10(b)) proteins in the prefrontal cortex of CUMS rats was higher than that in the control group. Compared to the CUMS group, IL-18 (*p* < 0.01Figure 10(a)) and IL-1*β* (*p* < 0.01Figure 10(b)) were significantly lower in the prefrontal cortex of rats in TCA group. Furthermore, FLU group exhibited a significant decrease in IL-18 (*p* < 0.01Figure 10(a)) and IL-1*β* (*p* < 0.01Figure 10(b)) in the prefrontal cortex.

### 3.10. TCA Blocks the NF-*κ*B Pathway of the Hippocampus at mRNA Level in CUMS Rats

**Figure 11:**
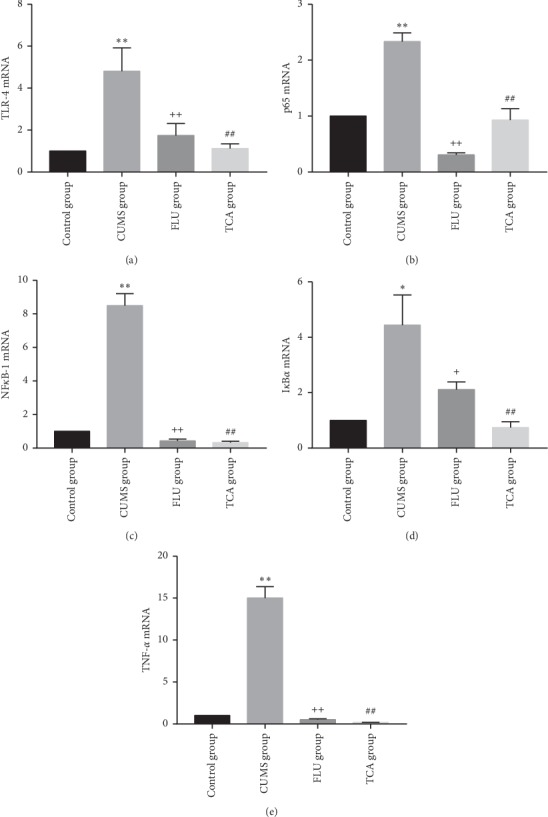
The effect of TCA on NF-*κ*B pathway of hippocampus at mRNA level. After 3 weeks of treatments, the relative expressions of mRNA of TLR-4 (a), p65 (b), NF-*κ*B-1 (c), I*κ*B*α* (d), and TNF-*α* (e) in hippocampus of rats were tested. ^*∗∗*^*p* < 0.01 comparison with the control group, ^++^*p* < 0.01 comparison with the CUMS group, ^##^*p* < 0.01 comparison with the CUMS group.

Relative expression of mRNAs of TLR-4, p65, NF-*κ*B-1, I*κ*B*α*, and TNF-*α* in hippocampus of rats was significantly higher in the CUMS group than that in the control group (*p* < 0.01, *p* < 0.01, *p* < 0.01, *p* < 0.01Figure 11(a) through Figure 11(e)). NF-*κ*B pathway mRNAs were downregulated by TCA and FLU after 3 weeks of treatment (*p* < 0.01, *p* < 0.01, *p* < 0.01, *p* < 0.01Figure 11(a) through Figure 11(e)).

### 3.11. TCA Blocks NF-*κ*B Pathway of the Hippocampus at Protein Level in CUMS Rats

**Figure 12:**
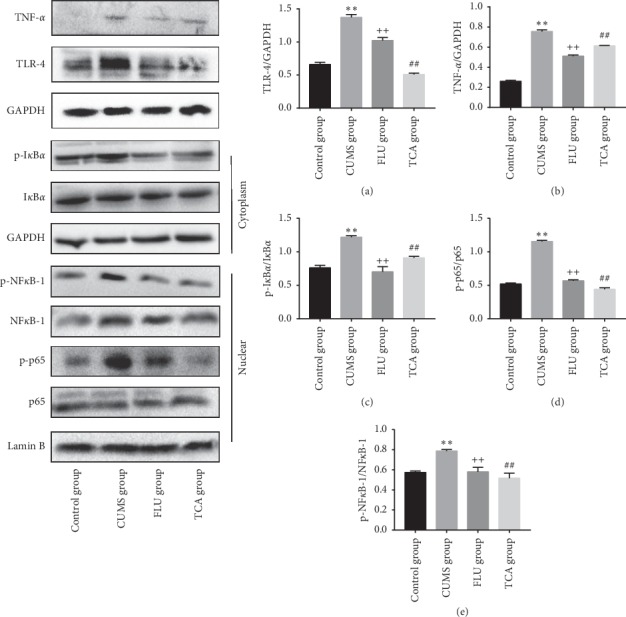
The effect of TCA on NF-*κ*B pathway of hippocampus at protein level. The protein expressions of TLR-4 (a), TNF-*α* (b), p-I*κ*B*α* (c), p-p65 (d), and p-NF-*κ*B-1 (e) in hippocampus of rats were tested after treatments. ^*∗∗*^*p* < 0.01 comparison with the control group, ^++^*p* < 0.01 comparison with the CUMS group, ^##^*p* < 0.01 comparison with the CUMS group.

The changes of NF-*κ*B pathway in hippocampus are shown in Figure 12. CUMS significantly upregulated the levels of TLR-4 (*p* < 0.01Figure 12(a)) and TNF-*α* (*p* < 0.01Figure 12(b)). The protein level of p-IKB*α* (*p* < 0.01Figure 12(c)) in cytoplasm and the protein levels of p-p65 (*p* < 0.01Figure 12(d)) and p-NF-*κ*B-1 (*p* < 0.01Figure 12(e)) in nucleus were also upregulated. After 3 weeks of treatment of TCA and FLU, respectively, the levels of TLR-4 (*p* < 0.01, *p* < 0.01Figure 12(a)) and TNF-*α* (*p* < 0.01, *p* < 0.01Figure 12(b)) were decreased. At the same time, the expression of p-IKB*α* (*p* < 0.01, *p* < 0.01Figure 12(c)) in cytoplasm and p-p65 (*p* < 0.01, *p* < 0.01Figure 12(d)) and p-NF-*κ*B-1 (*p* < 0.01, *p* < 0.01Figure 12(e)) in nucleus was downregulated significantly.

### 3.12. TCA Blocks the NLRP3 Inflammasome Activation of the Hippocampus in CUMS Rats

**Figure 13:**
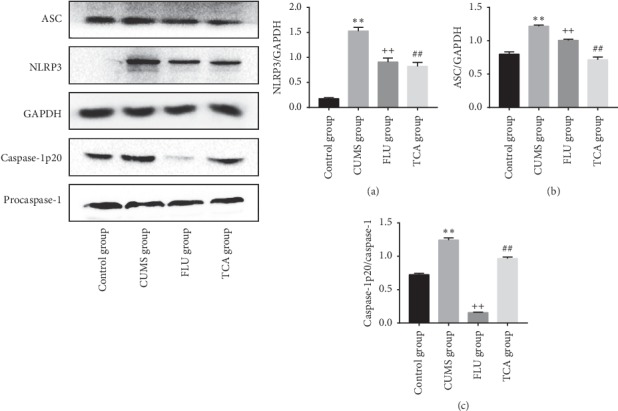
The effect of TCA on NLRP3 inflammasome of hippocampus. The expressions of NLRP3 (a), ASC (b), and caspase-1 p20 (c) in the hippocampus of rats were tested after treatments. ^*∗∗*^*p* < 0.01 comparison with the control group, ^++^*p* < 0.01 comparison with the CUMS group, ^##^*p* < 0.01 comparison with the CUMS group.

The effects of TCA on NLRP3 Inflammasome activation in the hippocampus are shown in Figure 13. The expressions of NLRP3, ASC, and caspase-1 p20 were markedly increased in the CUMS group compared to the control group (*p* < 0.01, *p* < 0.01, *p* < 0.01Figure 13(a) through Figure 13(c)). In contrast, after being treated with TCA and FLU, respectively, for 3 weeks, the NLRP3 Inflammasome activation was blocked (*p* < 0.01, *p* < 0.01, *p* < 0.01Figure 13(a) through Figure 13(c)).

### 3.13. TCA Reduces the Expression of Inflammatory Factors of the Hippocampus

**Figure 14:**
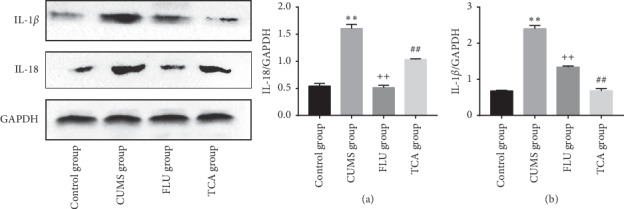
The effect of TCA on inflammatory factors of hippocampus. The expressions of IL-18 (a) and IL-1*β* (b) of rats in hippocampus were tested. ^*∗∗*^*p* < 0.01 comparison with the control group, ^++^*p* < 0.01 comparison with the CUMS group, ^##^*p* < 0.01 comparison with the CUMS group.

Figure 14 shows the effect of TCA on the hippocampal peripheral inflammatory factors in CUMS rats. The expression of IL-18 (*p* < 0.01Figure 14(a)) in the hippocampus of CUMS rats was more than that in the control group. TCA and FLU decreased the IL-18 protein expression within 3 weeks of treatment, respectively. A similar tendency was observed in the protein expression of IL-1*β* (*p* < 0.01Figure 14(b)).

### 3.14. TCA Reduces the Expression of Inflammatory Factors of Peripheral Blood

**Figure 15:**
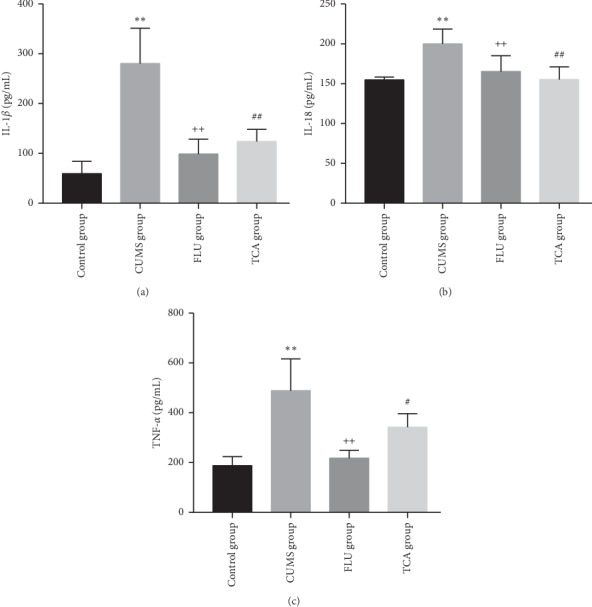
The effect of TCA on inflammatory factors in serum. The expressions of IL-1*β* (a), IL-18 (b), and TNF-*α* (c) in serum of rats were tested. ^*∗∗*^*p* < 0.01 comparison with the control group, ^++^*p* < 0.01 comparison with the CUMS group, ^#^*p* < 0.05 comparison with the CUMS group ^##^*p* < 0.01 comparison with the CUMS group.

As shown in Figure 15, the inflammatory factors in serum such as IL-1*β* (*p* < 0.01Figure 15(a)), IL-18 (*p* < 0.01Figure 15(b)), and TNF-*α* (*p* < 0.01Figure 15(c)) of rats were elevated after establishing the CUMS. The level of inflammatory factors (*p* < 0.01, *p* < 0.01 Figures 15(a) and 15(c)) was decreased after 3 weeks of FLU treatment. Compared to CUMS group, the IL-1*β* (*p* < 0.01Figure 15(a)), IL-18 (*p* < 0.01Figure 15(b)), and TNF-*α* (*p* < 0.05Figure 15(c)) were suppressed in the TCA group.

## 4. Discussion

In this study, a depression model of SD rats was induced by CUMS and used to explore the antidepressant-like effect and mechanism of TCA. The results showed that TCA and FLU could reverse the depression-like behaviors and neuroinflammation in rats with CUMS. The antidepressant-like mechanism of TCA might be due to its ability to reverse the morphological injury in the hippocampal pyramidal cells and the inhibition of NLRP3 inflammasome and NF-*κ*B pathway induced by CUMS in the prefrontal cortex and hippocampus of rats.

In China, *C. cassia* has been used to treat dyspepsia, blood circulation disturbances, and inflammatory disease for thousands of years. As a main active component of *C. cassia*, TCA has potent pharmacological effects, such as anti-neuroinflammation, anti-apoptosis, and anti-oxidant stress, etc. [23]. TCA is an effective treatment for neurodegenerative diseases, such as cerebral ischemic injury [21]. In addition, TCA inhibits the microglial activation and improves the neuronal survival against neuroinflammation in BV2 microglial cells with LPS stimulation [20].

CUMS is a well-validated model of depression. The depressive-like behaviors of the CUMS rats are similar to the clinical manifestations of depression that are caused by multiple stresses, including behavioral coping, motivational state, and cognitive symptoms [24]. As classical tests to validate depressive-like behaviors, SPT and FST are used to evaluate anhedonia [25] and behavioral despair [26]in rats. Our data showed that the CUMS rats exhibited depressive-like behaviors, which were evidenced by a significant decrease in body weight gain, increase in immobility time in FST, and a significant reduction in sucrose consumption compared to the rats in the control group. For the first time, we proved that TCA could reverse the behavioral disorder of the CUMS rats, which was evidenced by a significant increase in body weight gain, a decrease of immobility time in FST, and a significant increase in sucrose consumption compared to the rats in the control group. These results are similar to those of FLU. The antidepressant-like effect of TCA in the experimental environment was confirmed.

Hippocampus is mainly responsible for the emotional regulation, learning, and the formation and activation of situational memory, and it is also the main susceptible region of the brain affected by stress injury [27]. Atrophy of hippocampus and decrease of pyramidal cells are observed in patients with depression [28]. In this study, it was found that there was a severe pathological injury in the hippocampus of rats with CUMS that was characterized by a decrease in pyramidal cells in the hippocampal CA3 region, morphological changes, and disorder of the cell arrangement. The ultrastructure of pyramidal cells in the CA3 region showed traumatic changes, such as swelling of the mitochondria and decrease or even disappearance of the mitochondrial crest. FLU and TCA reversed the pathological injury of pyramidal cells in the hippocampal CA3 region of the CUMS rats, increased the number of pyramidal cells, and repaired the ultrastructure of the pyramidal cells.

TLR4 and NLRP3 are important pattern recognition receptors for detecting danger signals. TLR4, a member of the Toll-like family of proteins, can recognize various stimuli and activate related signal transduction pathways to induce the inflammatory response [29]. Mammalian NF-*κ*B represents a class of structurally related proteins, including RelA (p65), RelB, c-ReL, NF-*κ*B1 (p50), and NF-*κ*B2 (p52). P65 protein is encoded by RelA, and it contains a C-terminal transcription activation domain (TAD), which is necessary to induce target gene transcription. The inhibitory effect of I*κ*B on NF-*κ*B by retention in cytoplasm is mainly through the interaction with p65. NF-*κ*B1 gene encodes p105. The I*κ*B-like sequence of p105 is degraded to form mature NF-*κ*B1 (P50). P50 lacks the TAD and regulates the DNA binding activity of NF-*κ*B by forming RelA/p50 heterodimer [30]. P50 contains nuclear localization signal, which is the binding site of NF and DNA. The TAD of p65 can promote the binding of p50 to DNA and regulate the transcriptional activity of many target genes downstream [31]. Nuclear transduction pathway of NF-*κ*B includes classical and nonclassical ways [32]. The former is a regular regulatory pathway. Classical signaling proteins often exist as I*κ*B/p65/p50 inactive trimers in cytoplasm. Under the action of IKK, I*κ*B*α* is phosphorylated and then dissociated from p65/p50 dimer; p50 and p65 are dissociated into cytoplasm. Under the action of IKK and protein kinase A, p65 and p50 are phosphorylated, p-p65 and p-p50 into the nucleus, then exposing the nuclear localization sequence and binding to a specific DNA sequence [33, 34]. The phosphorylation level of I*κ*B*α*, p50, and p65 and the number of the p50 and p65 into the nucleus are the key to determine whether the classical NF-*κ*B signaling pathway is activated or not. The NF-*κ*B pathway is activated by TLR4 leading to initiation and magnification of the inflammatory response, and release of inflammatory factors and serious injury [35]. NLRP3 inflammasome is a pattern recognition receptor found in the cytoplasm. During the activation of NLRP3 inflammasome, NF-*κ*B pathway triggers pro-IL-1*β* and pro-IL-18 production. In addition, the microbial or risk-related molecular patterns such as reactive oxygen species (ROS), ATP release, and calcium overload activate NLRP3 inflammasome directly [36, 37]. As the core protein of the inflammasome, NLRP3 assembles ASC and procaspase-1 to form NLRP3 inflammasome [38]. IL-1*β* and IL-18 are released in large quantities by the promotion of the activated NLRP3 inflammasome. These oversecreted inflammatory cytokines form an “inflammatory waterfall” and induce the activation of immune inflammatory response.

The neuroinflammatory activation is used to explain the pathogenesis of depression, which is mediated by the NF-*κ*B pathway and NLRP3 inflammasome activation. TLR4 plays a central role in neuroinflammation induced by stress [39]. The stress induced by the increase in IL-1*β* in the hippocampal can be prevented by blocking TLR4 [40]. The activation of TLR4 is involved in the pathophysiological process of depression [41]. TLR4 is highly expressed in brain microglia, and excessive inflammation resulting from the activation of this pathway in the brain has been implicated in depressive disorders and neurodegenerative pathologies [42]. Broad inflammatory responses in hippocampus involve TLR4 and downstream inflammatory signaling induced by stresses. These stresses contribute to the susceptibility to depression-like behavior in mice [39]. Inflammation molecule, especially NF-*κ*B, levels in plasma are increased in patients with major depression [43]. NLRP3 inflammasome activation has been detected in the brain of CUMS animals [12, 44]. As one of the proinflammatory cytokines, IL-1*β* is implicated in stress, depression, and central nervous system (CNS) dysregulation [10]. The activation of the NF-*κ*B pathway and NLRP3 inflammasome activation might be a key node in the set of stress-induced risk signals. Therefore, this study was based on the activation of inflammation mediated by the NF-*κ*B pathway and NLRP3 inflammasome to explore the antidepressant-like effect of TCA. The inflammation levels in hippocampus and prefrontal cortex and the expression of serum inflammatory factors were examined.

Results indicate that the CUMS procedure significantly increased the NF-*κ*B pathway and NLRP3 inflammasome activation in hippocampus and prefrontal cortex. In the prefrontal cortex and hippocampus of CUMS rats, the relative expressions of NF-*κ*B pathway related mRNA, TLR-4, I*κ*B*α*, p65, NF-*κ*B-1, and TNF-*α* were increased, the protein levels of TLR-4 and TNF-*α* were increased, and the p-I*κ*B*α* level in cytoplasmic proteins and the expressions of p-p65 and p-NF-*κ*B-1 in the nucleus were also increased, indicating that CUMS activated the NF-*κ*B pathway. In addition, NLRP3 inflammasome activation was evidenced by the increase in the levels of NLRP3, ASC, and caspase-1 proteins in the prefrontal cortex and hippocampus. The downstream inflammatory factors (IL-1*β* and IL-18) were upregulated in different brain regions. These results are consistent with previous reports [45, 46].

FLU and TCA markedly inhibited the inflammatory response in hippocampus and prefrontal cortex of rats with CUMS by suppressing the NF-*κ*B pathway and NLRP3 inflammasome activation. The two drugs downregulate the relative expressions of NF-*κ*B pathway related mRNA (TLR-4, I*κ*B*α*, p65, NF-*κ*B-1, and TNF-*α*). They also decreased the p-I*κ*B*α* level in cytoplasmic proteins and the expressions of p-p65 and p-NF-*κ*B-1 in the nucleus significantly in different brain regions. FLU and TCA also have inhibition effects on TLR-4 and TNF-*α* in both hippocampus and prefrontal cortex, which might result from their inhibition of NF-*κ*B activation in the CUMS rats. The levels of NLRP3, ASC, and caspase-1 proteins after treatments were significantly decreased in FLU group and TCA group compared to the model group, indicating that FLU and TCA had significant inhibitory effect on activation of NLRP3 inflammasome. These inhibitory effects have been demonstrated in both the hippocampus and prefrontal cortex. Besides, FLU and TCA significantly inhibited the downstream inflammatory factors (IL-1*β* and IL-18) in different brain regions. In addition, the serum inflammatory factors such as IL-1*β*, IL-18, and TNF-*α* were downregulated by FLU and TCA. These inhibitory effects on the inflammatory factors might be related to the suppression of NF-*κ*B pathway and NLRP3 inflammasome.

In brief, these observations indicate that TCA acts as an antidepressant by suppressing the activation of the NF-*κ*B pathway and NLRP3 inflammasome in the prefrontal cortex and hippocampus of CUMS rats, at least, partially.

## 5. Conclusion

The potent antidepressant-like effect of TCA is presented for the first time, as evidenced by the increase in sucrose consumption and by the decrease in immobility time in FST of rats with CUMS. Our results suggest that the underlying pharmacological mechanisms of TCA might involve the inhibition of the inflammatory response by blocking the activation of NF-*κ*B pathway and NLRP3 inflammasome in prefrontal cortex and hippocampus of rats with CUMS. These results reveal the mechanism of the antidepressant-like effect of TCA.

## Figures and Tables

**Table 1:** Stressors of CUMS.

Stressors	Duration	times
4°C ice water swimming	5 min	5
Cage tilting (45°)	12 h	4
Overnight lighting	36 h	3
Wet pad (1000 ml water per cage)	15 h	4
Food deprivation	24 h	5
Water deprivation	24 h	5
Clamping tail	5 min	4
Crowding	3 h	5
Cage shaking	5 min	3
Vinegar stimulation (20 ml vinegar per cage)	24 h	4

## Data Availability

The data required to reproduce these findings cannot be shared at this time as the data also forms part of our ongoing subject.

## References

[1] Vos T., Allen C., Arora M. (2016). Global, regional, and national incidence, prevalence, and years lived with disability for 310 diseases and injuries, 1990–2015. *Lancet*.

[2] WHO (2008). *The Global Burden of Disease: 2004 Update*.

[3] Saba M., Somnath C., Emese V., Ajay T., Vikram P., Bedirhan U. (2007). Depression, chronic diseases, and decrements in health: results from the world health surveys. *The Lancet*.

[4] John R. A., Trivedi M. H., Wisniewski S. R. (2006). Acute and longer-term outcomes in depressed outpatients requiring one or several treatment steps: a STAR∗D report. *American Journal of Psychiatry*.

[5] Miller A. H., Raison C. L. (2016). The role of inflammation in depression: from evolutionary imperative to modern treatment target. *Nature Reviews Immunology*.

[6] Dantzer R., O’Connor J. C., Lawson M. A., Kelley K. W. (2011). Inflammation-associated depression: from serotonin to kynurenine. *Psychoneuroendocrinology*.

[7] Malhi G. S., Mann J. J. (2018). Depression. *The Lancet*.

[8] Martinon F., Burns K., Tschopp J. (2002). The inflammasome: a molecular platform triggering activation of inflammatory caspases and processing of proIL-*β*. *Molecular Cell*.

[9] Kaufmann F. N., Costa A. P., Ghisleni G. (2017). Nlrp3 inflammasome-driven pathways in depression: clinical and preclinical findings. *Brain Behavior & Immunity*.

[10] Iwata M., Ota K. T., Duman R. S. (2013). The inflammasome: pathways linking psychological stress, depression, and systemic illnesses. *Brain, Behavior, and Immunity*.

[11] Alcocer-Gómez E., de Miguel M., Casas-Barquero N. (2014). NLRP3 inflammasome is activated in mononuclear blood cells from patients with major depressive disorder. *Brain, Behavior, and Immunity*.

[12] Pan Y., Chen X.-Y., Zhang Q.-Y., Kong L.-D. (2014). Microglial NLRP3 inflammasome activation mediates IL-1*β*-related inflammation in prefrontal cortex of depressive rats. *Brain, Behavior, and Immunity*.

[13] Su W.-J., Zhang Y., Chen Y. (2017). NLRP3 gene knockout blocks NF-*κ*B and MAPK signaling pathway in CUMS-induced depression mouse model. *Behavioural Brain Research*.

[14] Du R.-H., Tan J., Sun X.-Y., Lu M., Ding J.-H., Hu G. (2016). Fluoxetine inhibits NLRP3 inflammasome activation: implication in depression. *International Journal of Neuropsychopharmacology*.

[15] Lee S.-C., Wang S.-Y., Li C.-C., Liu C.-T. (2018). Anti-inflammatory effect of cinnamaldehyde and linalool from the leaf essential oil of *Cinnamomum osmophloeum* Kanehira in endotoxin-induced mice. *Journal of Food and Drug Analysis*.

[16] Chew E.-H., Nagle A. A., Zhang Y. (2010). Cinnamaldehydes inhibit thioredoxin reductase and induce Nrf2: potential candidates for cancer therapy and chemoprevention. *Free Radical Biology and Medicine*.

[17] Subash-Babu P., Alshatwi A. A., Ignacimuthu S. (2014). Beneficial antioxidative and antiperoxidative effect of cinnamaldehyde protect streptozotocin-induced pancreatic *β*-cells damage in wistar rats. *Biomolecules & Therapeutics*.

[18] Khare P., Jagtap S., Jain Y. (2016). Cinnamaldehyde supplementation prevents fasting-induced hyperphagia, lipid accumulation, and inflammation in high-fat diet-fed mice. *Biofactors*.

[19] Pyo J.-H., Jeong Y.-K., Yeo S. (2013). Neuroprotective effect of trans-cinnamaldehyde on the 6-hydroxydopamine-induced dopaminergic injury. *Biological & Pharmaceutical Bulletin*.

[20] Fu Y., Wu Z., Dong X. (2017). *trans*-Cinnamaldehyde inhibits microglial activation and improves neuronal survival against neuroinflammation in BV2 microglial cells with lipopolysaccharide stimulation. *Evidence-Based Complementray and Alternative Medicine*.

[21] Chen Y.-F., Wang Y.-W., Huang W.-S. (2016). Trans-cinnamaldehyde, an essential oil in cinnamon powder, ameliorates cerebral ischemia-induced brain injury via inhibition of neuroinflammation through attenuation of iNOS, COX-2 expression and NF*κ*-B signaling pathway. *NeuroMolecular Medicine*.

[22] Ka S.-M., Kuoping Chao L., Lin J.-C. (2016). A low toxicity synthetic cinnamaldehyde derivative ameliorates renal inflammation in mice by inhibiting NLRP3 inflammasome and its related signaling pathways. *Free Radical Biology and Medicine*.

[23] Qi X., Zhou R., Liu Y. (2016). Trans-cinnamaldehyde protected PC12 cells against oxygen and glucose deprivation/reperfusion (OGD/R)-induced injury via anti-apoptosis and anti-oxidative stress. *Molecular & Cellular Biochemistry*.

[24] Liu X.-J., Li Z.-Y., Li Z.-F. (2012). Urinary metabonomic study using a CUMS rat model of depression. *Magnetic Resonance in Chemistry*.

[25] Martynhak B. J., Correia D., Morais L. H. (2011). Neonatal exposure to constant light prevents anhedonia-like behavior induced by constant light exposure in adulthood. *Behavioural Brain Research*.

[26] Castagné V., Moser P., Roux S., Porsolt R. D. (2011). Rodent models of depression: forced swim and tail suspension behavioral despair tests in rats and mice. *Current Protocols in Neuroscience*.

[27] Diamond D. M., Park C. R., Campbell A. M., Woodson J. C. (2005). Competitive interactions between endogenous LTD and LTP in the hippocampus underlie the storage of emotional memories and stress-induced amnesia. *Hippocampus*.

[28] Taylor W. D., Mcquoid D. R., Payne M. E., Zannas A. S., Macfall J. R., Steffens D. C. (2014). Hippocampus atrophy and the longitudinal course of late-life depression. *The American Journal of Geriatric Psychiatry*.

[29] Liu S. P., Li X. Y., Li Z. (2012). Octanoylated ghrelin inhibits the activation of the palmitic acid-induced TLR4/NF-*κ*B signaling pathway in THP-1 macrophages. *ISRN Endocrinology*.

[30] Zhang Q., Lenardo M. J., Baltimore D. (2017). 30 years of NF-*κ*B: a blossoming of relevance to human pathobiology. *Cell*.

[31] Perkins N. D. (2012). The diverse and complex roles of NF-*κ*B subunits in cancer. *Nature Reviews Cancer*.

[32] Morotti A., Crivellaro S., Panuzzo C., Carrà G., Guerrasio A., Saglio G. (2017). I*κ*B-*α*: at the crossroad between oncogenic and tumor-suppressive signals. *Oncology Letters*.

[33] Postler T. S., Ghosh S. (2015). Bridging the gap: a regulator of NF-*κ*B linking inflammation and cancer. *Journal of Oral Biosciences*.

[34] Krappmann D., Vincendeau M. (2016). Mechanisms of NF-*κ*B deregulation in lymphoid malignancies. *Seminars in Cancer Biology*.

[35] Chen G., Shi J., Hu Z., Hang C. (2008). Inhibitory effect on cerebral inflammatory response following traumatic brain injury in rats: a potential neuroprotective mechanism of N-acetylcysteine. *Mediators of Inflammation*.

[36] Heid M. E., Keyel P. A., Kamga C., Shiva S., Watkins S. C., Salter R. D. (2013). Mitochondrial reactive oxygen species induces NLRP3-dependent lysosomal damage and inflammasome activation. *The Journal of Immunology*.

[37] Geun-Shik L., Naeha S., Kim A. I. (2012). The calcium-sensing receptor regulates the NLRP3 inflammasome through Ca^2+^ and cAMP. *Nature*.

[38] Py B. F., Kim M.-S., Vakifahmetoglu-Norberg H., Yuan J. (2013). Deubiquitination of NLRP3 by BRCC3 critically regulates inflammasome activity. *Molecular Cell*.

[39] Cheng Y., Pardo M., Armini R. d. S. (2016). Stress-induced neuroinflammation is mediated by GSK3-dependent TLR4 signaling that promotes susceptibility to depression-like behavior. *Brain, Behavior, and Immunity*.

[40] Weber M. D., Frank M. G., Sobesky J. L., Watkins L. R., Maier S. F. (2013). Blocking toll-like receptor 2 and 4 signaling during a stressor prevents stress-induced priming of neuroinflammatory responses to a subsequent immune challenge. *Brain, Behavior, and Immunity*.

[41] Dantzer R., Kelley K. W. (2007). Twenty years of research on cytokine-induced sickness behavior. *Brain, Behavior, and Immunity*.

[42] Hines D. J., Choi H. B., Hines R. M., Phillips A. G., Macvicar B. A. (2013). Prevention of LPS-induced microglia activation, cytokine production and sickness behavior with TLR4 receptor interfering peptides. *PLoS One*.

[43] Pace T. W. W., Mletzko T. C., Alagbe O. (2006). Increased stress-induced inflammatory responses in male patients with major depression and increased early life stress. *American Journal of Psychiatry*.

[44] Lu M., Yang J.-Z., Geng F., Ding J.-H., Hu G. (2014). Iptakalim confers an antidepressant effect in a chronic mild stress model of depression through regulating neuro-inflammation and neurogenesis. *The International Journal of Neuropsychopharmacology*.

[45] Jia K.-K., Zheng Y.-J., Zhang Y.-X. (2017). Banxia-houpu decoction restores glucose intolerance in CUMS rats through improvement of insulin signaling and suppression of NLRP3 inflammasome activation in liver and brain. *Journal of Ethnopharmacology*.

[46] Zhang Y., Liu L., Liu Y.-Z. (2015). NLRP3 inflammasome mediates chronic mild stress-induced depression in mice via neuroinflammation. *International Journal of Neuropsychopharmacology*.

